# A Case of Metastatic Non-small Cell Lung Adenocarcinoma and Metachronous Primary Hepatocellular Carcinoma

**DOI:** 10.7759/cureus.20185

**Published:** 2021-12-05

**Authors:** Fady Sidhom, Ahmed Ali, Mohd Elmugtaba Ibrahim

**Affiliations:** 1 Internal Medicine, Howard University Hospital, Washington, USA; 2 Medical Oncology, Howard University Hospital, Washington, USA

**Keywords:** transarterial embolization, next generation sequencing (ngs), multiple primary malignant neoplasm, non-small cell lung carcinoma (nsclc), hepatocellular carcinoma (hcc), multiple primary neoplasms

## Abstract

Multiple primary malignant neoplasms (MPMNs) are generally defined as the co-occurrence of primary neoplasms of distinct histology in the same individual. Second and higher-order primary malignancies now comprise about 18% of all cancer incidence in the United States. Specifically in female cancer survivors, the incidence ratio of developing multiple primary cancers (MPCs) is 1.2 to 1.6. Patients with lung cancer are at higher risk to develop a second malignancy compared to the general population. However, the coexistence of non-small cell lung adenocarcinoma and primary hepatocellular carcinoma (HCC) is not described in the literature. Here we describe a rare case of a 69-year-old female with non-small cell lung adenocarcinoma with vertebral metastasis that developed primary HCC.

## Introduction

Multiple primary malignant neoplasms (MPMNs) are categorized as ​​synchronous if the tumors are diagnosed within six months and metachronous if diagnosed more than six months apart. Based on the definition by the International Association of Cancer Registries, our patient was classified as metachronous. Due to better screening and detection, as well as improved therapies leading to longer life expectancy in cancer patients, more and more cases of MPMNs are being identified. Patients with a history of malignancy have a 14% higher chance of developing a secondary cancer compared to the general population [1]. Specifically, in patients with lung cancers, the most common second cancers are those of the lung followed by colorectal and bladder cancers [2], but our patient presented with a rare secondary cancer in the form of hepatocellular carcinoma (HCC). To our knowledge, a case of primary non-small cell adenocarcinoma of the lung and primary HCC has never been described in the literature.

## Case presentation

**Figure 1:**
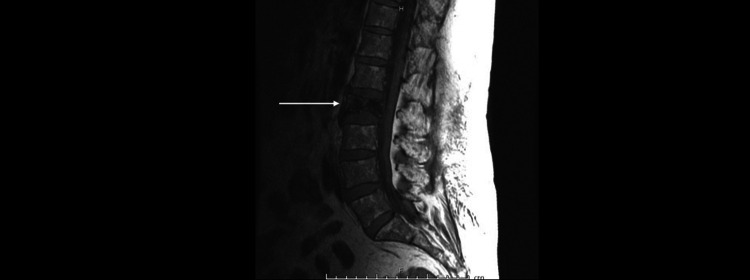
MRI lumbar spine with contrast. The white arrow shows L2 increased heterogeneity and an overall decrease in signal intensity.

**Figure 2:**
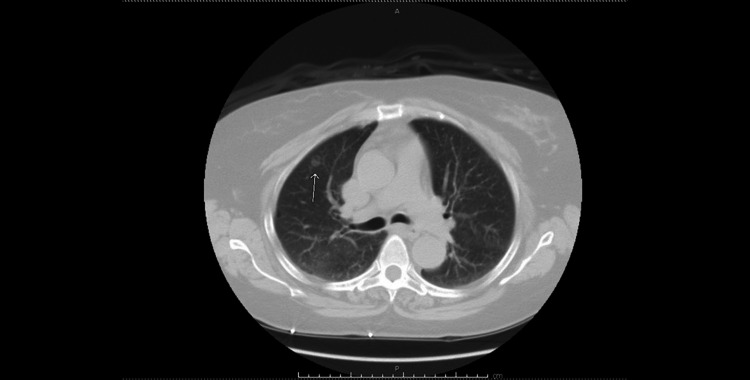
CT chest without contrast. White arrow showing right upper lobe nodule that was biopsied.

**Figure 3:**
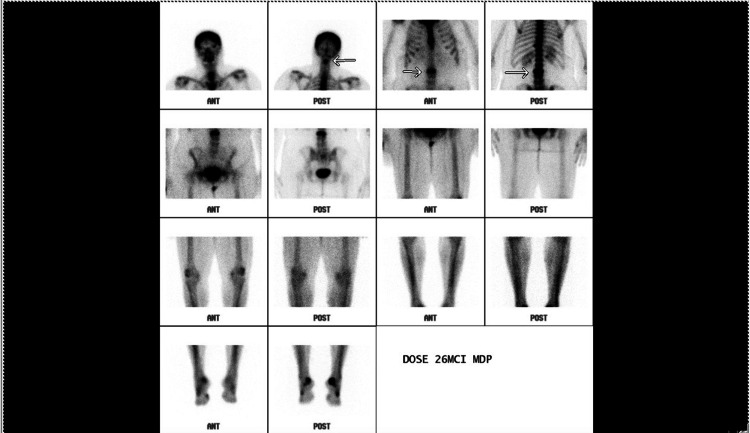
Bone scan showing solitary metastasis in the L3 vertebral body.

**Figure 4:**
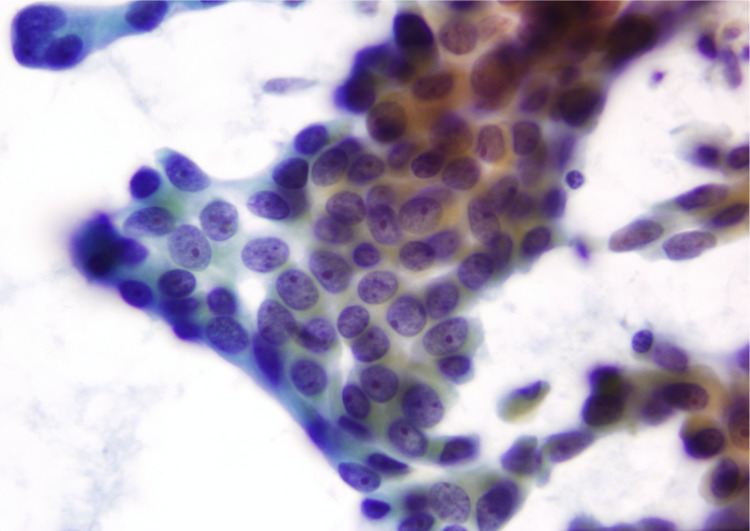
Fine needle aspiration of the lumbar spine; syncytial groupings and single-lying tumor cells with occasional nuclear crowding and overlapping.

**Figure 5:**
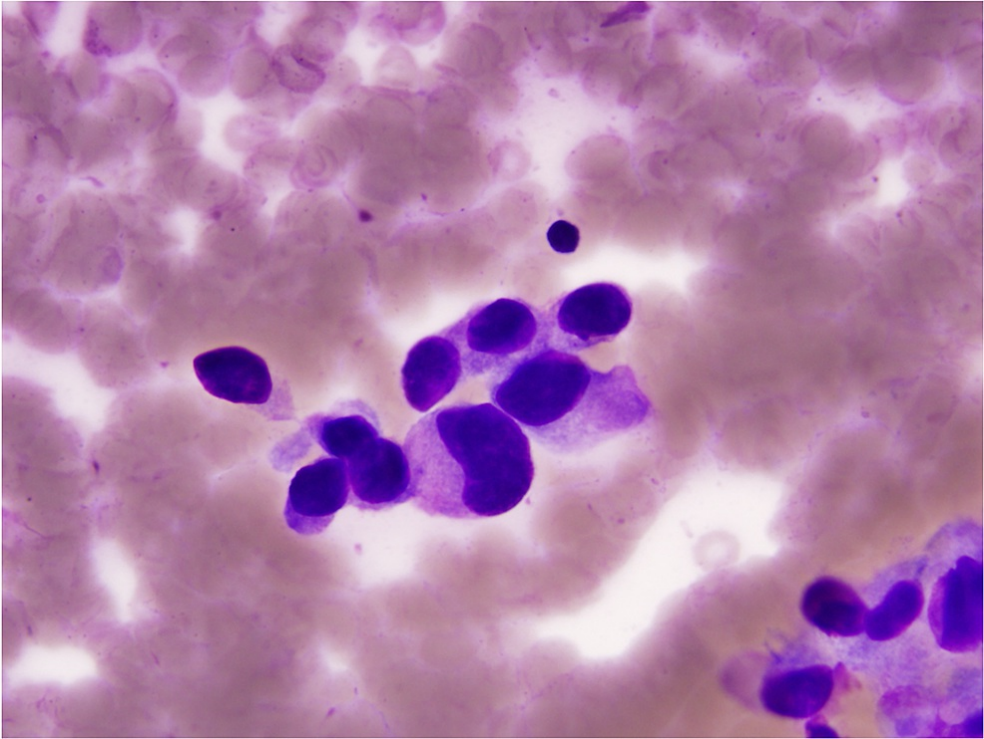
Fine needle aspiration of the right upper lobe lung; tumor cells with high N/C ratios, enlarged hyperchromatic and pleomorphic nuclei. N/C ratio: Nuclear-cytoplasmic ratio.

We present a case of a 69-year-old female with an established history of hepatitis C and cirrhosis, who initially presented with the complaint of worsening back pain for a one-year duration. An X-ray of the lumbar spine was initially obtained that was significant for vertebral body sclerosis and recommended an MRI for further evaluation. MRI lumbar spine showed increased heterogeneity of L2 vertebrae, decrease in signal intensity compared to the remaining vertebra, and marrow replacement (Figure 1). The MRI findings were non-specific findings for metastasis. CT chest imaging was significant for a 1 cm right lung nodule and a 1.8 x 1.7 cm left lung nodule (Figure 2). A bone scan revealed solitary metastasis in the L3 vertebral body (Figure 3). The patient underwent a biopsy of the right upper nodule which was positive for adenocarcinoma (Figure 4). The patient also underwent an L2 vertebral lesion biopsy showing metastatic adenocarcinoma consistent with primary lung cancer (Figure 5). The patient received radiation therapy of the spine from L1 to L3 using three-dimensional conformal radiotherapy. Thyroid transcription factor-1 (TTF1) expression confirmed primary adenocarcinoma originating in the lung, and an epidermal growth factor receptor (EGFR) mutation was also found. The patient was subsequently started on gefitinib and denosumab. 

**Figure 6:**
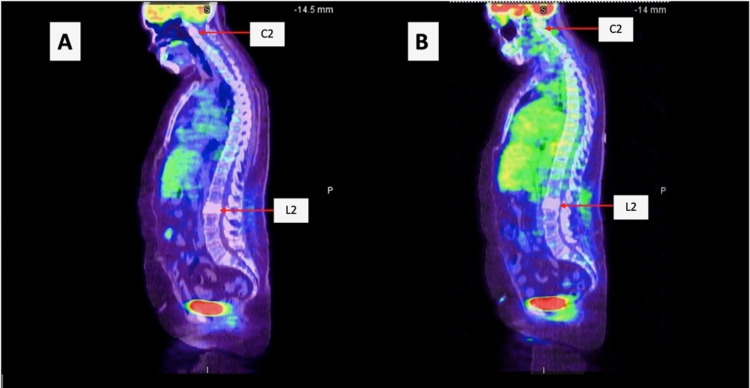
A. Initial PET scan showing diffuse sclerotic lesion in C2 and L2 vertebrae and no other hypermetabolic areas. B. Follow-up PET scan showing stable sclerotic lesions and no hypermetabolic activity. PET: Positron emission tomography.

**Figure 7:**
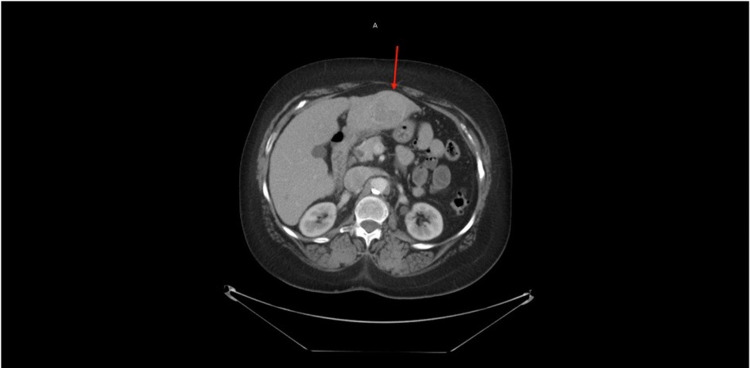
CT chest without contrast showing left hepatic lobe lesion with heterogeneous enhancement.

**Figure 8:**
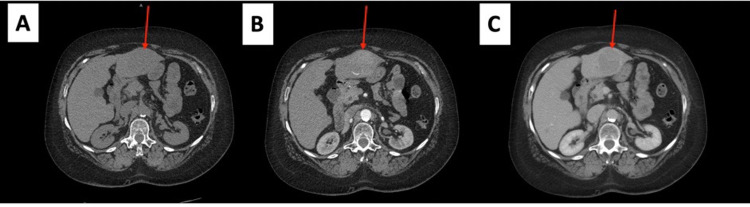
Triple phase CT scan of the liver. A. Non-contrast image showing isodense mass (arrow) in the left lobe of the liver. B. Arterial phase image showing hypodense/heterogeneous mass. C. Fast washout of the tumor mass in the portal venous phase.

A positron emission tomography (PET) scan was done to evaluate for progression and response to treatment showed a diffuse sclerotic lesion in L2 and C2 vertebral bodies but no other hypermetabolic area (Figure 6). A repeat PET scan was done the following year and showed no hypermetabolic activity with a stable sclerotic lesion (Figure 6). A surveillance CT chest, abdomen, and pelvis done the following year showed multiple lung nodules as well as a liver mass, possibly hemangioma (Figure 7). A triple-phase CT abdomen confirmed a liver mass with enhancement (Figure 8). A liver biopsy was done and confirmed HCC with elevated alpha-fetoprotein (AFP) and carcinoembryonic antigen (CEA). Liquid biopsy results sent for next-generation sequencing (NGS) showed gatekeeper mutation of the T790M missense variant (exon 20). The patient was subsequently switched to osimertinib. The patient was referred to surgical oncology but was deemed a non-surgical candidate due to her history of hepatitis C and cirrhosis. Shortly after, the patient underwent a transarterial radioembolization (TARE) treatment of her HCC with a good response. The patient continues to follow up regularly with oncology.

## Discussion

MPMNs were first described in 1932 and have become an increasingly important factor in oncology practice today [3]. The incidence of MPMN is approximately 18% in the United States. MPMNs present various treatment challenges for oncologists since therapies often have to be adapted and this often comes with an uncertain prognosis for patients. Within the world of precision oncology, next-generation sequencing methods that allow for the sequencing of entire genomes have become a critical part of the management of MPMNs. NGS can identify actionable mutations such as EGFR in NSCLC. Two major activating mutations in EGFR are in-frame deletion in exon 19 (del-19) and the L858R substitution in exon 21. These mutations account for 85% of all clinically important mutations related to EGFR-tyrosine kinase inhibitors (TKIs) sensitivity [4]. Other mutations have been discovered in EGFR exons 18-21, however, these rare mutations are not fully described [5]. Osimertinib, the agent prescribed for our patient, is an oral third-generation irreversible EGFR-TKI that selectively inhibits both EGFR-TKI-sensitizing and EGFR nonT790M resistance mutations [6]. Osimertinib had significantly greater efficacy than platinum therapy plus pemetrexed in patients with T790M-positive advanced NSCLC [7].

Our patient was a candidate for TARE, which is an evolving experimental treatment for unresectable HCC. A systematic review done by Rognoni C et al. showed that the overall survival (OS) rate in patients with intermediate-stage HCC was higher in patients with advanced disease, even with appreciable liver function on account of the invasion of the tumor in the portal vein. That notwithstanding, a mixture of intermediate and advanced cases resulted in a higher OS than in advanced alone. They also concluded TARE has a wide variability of side effects in terms of liver decompensation and is primarily dependent on the Child-Pugh score, number of procedures, and the duration of treatment. Based on their retrospective study, TARE is a valuation treatment option for patients with intermediate and advanced HCC [8].

## Conclusions

In conclusion, we present a rare case of MPMN with non-small cell adenocarcinoma and metachronous HCC. NGS was used to identify an EGFR mutation leading to osimertinib therapy. Our patient also received experimental TARE therapy for the management of her unresectable HCC. MPMNs present various challenges for oncologists and additional studies are required to evaluate novel therapies in the management of MPMNs.

## References

[1] (2006). New Malignancies Among Cancer Survivors: SEER Cancer Registries, 1973-2000. https://seer.cancer.gov/archive/publications/mpmono/MPMonograph_complete.pdf.

[2] Vogt A, Schmid S, Heinimann K, Frick H, Herrmann C, Cerny T, Omlin A (2017). Multiple primary tumours: challenges and approaches, a review. ESMO Open.

[3] Warren Shields, Ehrenreich Theodore (1944). Multiple primary malignant tumors and susceptibility to cancer. Cancer Res.

[4] Lynch TJ, Bell DW, Sordella R (2004). Activating mutations in the epidermal growth factor receptor underlying responsiveness of non-small-cell lung cancer to gefitinib. N Engl J Med.

[5] Wu JY, Yu CJ, Chang YC, Yang CH, Shih JY, Yang PC (2011). Effectiveness of tyrosine kinase inhibitors on "uncommon" epidermal growth factor receptor mutations of unknown clinical significance in non-small cell lung cancer. Clin Cancer Res.

[6] Soria JC, Ohe Y, Vansteenkiste J (2018). Osimertinib in untreated EGFR-mutated advanced non-small-cell lung cancer. N Engl J Med.

[7] Mok TS, Wu Y-L, Ahn M-J (2017). Osimertinib or platinum-pemetrexed in EGFR T790M-positive lung cancer. N Engl J Med.

[8] Rognoni C, Ciani O, Sommariva S, Facciorusso A, Tarricone R, Bhoori S, Mazzaferro V (2016). Trans-arterial radioembolization in intermediate-advanced hepatocellular carcinoma: systematic review and meta-analyses. Oncotarget.

